# Profiling the Salivary microbiome of the Qatari population

**DOI:** 10.1186/s12967-020-02291-2

**Published:** 2020-03-14

**Authors:** Selvasankar Murugesan, Sara Fahad Al Ahmad, Parul Singh, Marwa Saadaoui, Manoj Kumar, Souhaila Al Khodor

**Affiliations:** 1 Research Department, Sidra Medicine, Doha, Qatar; 2 College of Health Sciences, Qatar University, Doha, Qatar

**Keywords:** 16S rRNA gene sequencing, Saliva, Dysbiosis, Qatari, Oral health, Qatar Biobank

## Abstract

**Background:**

The role of the human microbiome in human health and disease has been studied in various body sites. However, compared to the gut microbiome, where most of the research focus is, the salivary microbiome still bears a vast amount of information that needs to be revealed. This study aims to characterize the salivary microbiome composition in the Qatari population, and to explore specific microbial signatures that can be associated with various lifestyles and different oral conditions.

**Materials and methods:**

We characterized the salivary microbiome of 997 Qatari adults using high-throughput sequencing of the V1–V3 region of the 16S rRNA gene.

**Results:**

In this study, we have characterized the salivary microbiome of 997 Qatari participants. Our data show that *Bacteroidetes*, *Firmicutes*, *Actinobacteria* and *Proteobacteria* are the common phyla isolated from the saliva samples, with Bacteroidetes being the most predominant phylum. *Bacteroidetes* was also more predominant in males versus females in the study cohort, although differences in the microbial diversity were not statistically significant. We also show that, a lower diversity of the salivary microbiome is observed in the elderly participants, with *Prevotella* and *Treponema* being the most significant genera. In participants with oral conditions such as mouth ulcers, bleeding or painful gum, our data show that *Prevotella* and *Capnocytophaga* are the most dominant genera as compared to the controls. Similar patterns were observed in participants with various smoking habits as compared to the non-smoking participants. Our data show that *Streptococcus* and *Neisseria* are more dominant among denture users, as compared to the non-denture users. Our data also show that, abnormal oral conditions are associated with a reduced microbial diversity and microbial richness. Moreover, in this study we show that frequent coffee drinkers have higher microbial diversity compared to the non-drinkers, indicating that coffee may cause changes to the salivary microbiome. Furthermore, tea drinkers show higher microbial richness as compared to the non-tea drinkers.

**Conclusion:**

This is the first study to assess the salivary microbiome in an Arab population, and one of the largest population-based studies aiming to the characterize the salivary microbiome composition and its association with age, oral health, denture use, smoking and coffee-tea consumption.

## Background

The human microbiota is the collection of a wide array of microorganisms such as bacteria, archaea, fungi and viruses that inhabit various body sites including skin, saliva and the gut [1]. The microbiome, defined as the collection of microbiota and their genes, plays an important role in human health and disease [2]. The development in the field of sequencing and bioinformatic tools in the last decade, has brought an unprecedented attraction to the microbiome field [3]. The microbiome composition varies from one person to another, as well as across different body sites [4].

Saliva is a biofluid secreted by the major and minor salivary glands [5]. It contains several components such as electrolytes, proteins, immunoglobulins, enzymes and microbes [6]. The main role of the saliva is to protect the mucus from pathogens, to maintain tooth integrity in addition to its role in taste and digestion [7]. Being highly available, saliva is considered as an easy to collect sample that does not require hospitalization or special preparation [8, 9].

Located at the opening of the gastrointestinal tract, the oral cavity provides a convenient, accessible site for collecting and analyzing microbial samples in the saliva [10]. It is also worth noting that, the salivary microbiome mirrors the gut microbiome in terms of complexity and diversity [10]. The salivary microbiome exhibits long-term stability and does not fluctuate according to the circadian rhythm, indicating that the time of the saliva sampling is not critical [11–16]. Collection of saliva can be achieved by several methods, including spitting, swabbing and draining [17], and the method of collection exerts a minimal effect on the extraction of microbial DNA [18]. A healthy adult human mouth hosts a complex and resilient ecosystem of hundreds of different microbial species [19]. These microbes reside in different sites of the oral cavity, which is mainly composed of a soft mucosa that is constantly shedding, and a hard surface which comprises the teeth [20]. The saliva is a representing constituent of both locations but more profoundly of the soft mucosa [20]. According to many studies, the salivary microbiome consists of more than 700 species mainly belonging to the *Bacteroidetes*, *Firmicutes*, *Proteobacteria*, *Actinobacteria* and *Fusobacteria* phyla [21–24]. *Streptococcus*, *Prevotella*, *Neisseria*, *Haemophilus*, *Porphyromonas* and *Rothia* are the common genera members of salivary microbiome observed in healthy adults [25–27]. The salivary microbiome composition is influenced by several pre and post-natal factors including host genetics, the mode of delivery at birth; the method of infant feeding; teeth eruption, the use of medications, especially antibiotics; smoking, intraoral pH, oral hygiene and diet among others [28]. The salivary microbiome plays a major role in regulating the immune-inflammatory balance in the host [29]. In a large American cohort study, Wu et al. compared the salivary microbiome composition in current smokers and non-smokers [30]. They observed that the salivary microbiome of smokers reflected a decrease in the abundance of the phylum *Proteobacteria*, and in *Capnocytophaga*, *Peptostreptococcus* and *Leptotrichia* genera; while the genera *Atopobium* and *Streptococcus* were found to be elevated in smokers compared to non-smokers [30]. Another study examined the oral microbiome of smokers and non-smokers in addition to the levels of cytokines in saliva samples, where they found that smoking altered the cytokine levels and the salivary microbiome composition [31].

Dysbiosis of the oral microbiome has been implicated in various oral disorders such as periodontitis, tooth decay or loss of teeth, where it promotes pathogenic bacterial growth and enables the dissemination of the oral bacteria systemically [32]. Several studies were conducted in order to assess the microbiome composition and its role in dental and periodontal health, and showed that, healthy individuals have a greater microbial diversity and a greater abundance of *Neisseria*, *Haemophilus*, and *Fusobacterium*. This is in contrast to individuals who suffer from dental caries, where Streptococcus was the most abundant genus detected [33, 34]. In a comparative study of healthy Finnish adults with and without caries, *Corynebacterium*, *Fusobacterium*, *Capnocytophaga*, *Porphyromonas*, *Prevotella*, and *Leptotrichia* were significantly more abundant in healthy volunteers as compared to those with dental caries [35].

*Porphyromonas gingivalis*, *Tannerella forsythia*, *Treponema denticola*, *Prevotella intermedia* and *Aggregatibacter actinomycetemcomitans* were shown to be higher in Moroccan patients with periodontitis [36] and *P. gingivalis*, *P. intermedia*, *T. forsythia* and *Fretibacterium* were higher in Japanese patients with periodontitis [37, 38]. Moreover, oral microbial dysbiosis is usually observed in patients with systemic diseases such as obesity, diabetes, cancer, rheumatoid arthritis, Parkinson’s disease, type 2 diabetes (T2D) and cardiovascular diseases among others [39–45].

Based on its potential role in health and disease, the salivary microbiome harbors a great potential for being used as a health monitor or disease diagnostic tool. However, the degree of variation at the population level has been assessed in very few studies [12, 46, 47], none of them reflecting the Arab population. The aim of this study is to characterize the salivary microbiome of the Qatari population and to assess the role of gender, age, oral health, smoking and some dietary habits in the salivary microbiome composition.

## Results

### Demographic and clinical parameters

We determined the bacterial compositions in the saliva samples of 997 Qatari adults aged ≥ 18 years using 16S rRNA gene sequencing. The subjects’ demographic and clinical characteristics including oral hygiene practices are summarized in Table 1.

**Table 1:** Demographic status of studied Qatari population

Category	Male	Female	Total
Gender	442	555	997
Age^a^	38.3 ± 11.9	39.3 ± 12.1	38.2 ± 12.0
Age group			
Adults (18 ≥ age ≤ 65)	434 (98.2%)	545 (98.2%)	979 (98.2%)
Elderly (age > 65)	8 (1.8%)	10 (1.8%)	18 (1.8%)
Oral health status			
Bleeding gum			
No	398 (90.0%)	515 (92.8%)	913 (91.6%)
Yes	44 (10%)	40 (7.20%)	84 (8.4%)
Mouth ulcer			
No	419 (94.8%)	541 (97.5%)	960 (96.3%)
Yes	23 (5.2%)	14 (2.5%)	37 (3.7%)
Painful gum			
No	400 (90.5%)	516 (93.0%)	916 (91.9%)
Yes	42 (9.5%)	39 (7.0%)	81 (8.1%)
Loose teeth			
No	422 (95.5%)	537 (96.8%)	959 (96.2%)
Yes	20 (4.5%)	18 (3.2%)	38 (3.8%)
Denture			
No	387 (87.6%)	474 (85.4%)	861 (86.4%)
Yes	55 (12.4%)	81 (14.6%)	136 (13.6%)
Smoking status			
Non-smokers	210 (47.5%)	523 (94.2%)	733 (73.5%)
Smokers	232 (52.5%)	32 (5.8%)	264 (26.5%)
Coffee drinking			
Coffee	418 (94.6%)	469 (84.5%)	887 (89.0%)
No coffee	24 (5.4%)	86 (15.5%)	110 (11.0%)
Tea drinking			
Tea	363 (82.1%)	405 (73.0%)	768 (77.0%)
No tea	79 (17.9%)	150 (27.0%)	229 (23.0%)

^a^Age: (years, mean ± standard deviation)

### The salivary microbiome of the Qatari population

From the analysis of 16S rRNA gene data, 10 different bacterial phyla and 112 genera were identified in the saliva samples collected form the Qatari participants included in this study. *Bacteroidetes* (65.9%), *Firmicutes* (15.8%), *Proteobacteria* (2.7%), *Fusobacteria* (0.7%) and *Saccharibacteria* (0.3%) were the top five phyla observed covering about 85% of the salivary microbiome composition (Additional file 1: Figure S1A). At the genus levels, our data show that a total of 13 genera (*Prevotella*, *Porphyromonas*, *Streptococcus*, *Veillonella*, *Capnocytophaga, Haemophilus*, *Gemella*, *Alloprevotella*, *Granulicatella*, *Camphylobacter*, *Leptotrichia*, *Megasphaera* and *Neisseria*) were commonly represented in all participants covering 85% of the salivary microbiome profiles (Additional file 1: Figure S1B). We then compared the salivary microbiome composition of the Qatari population to the salivary microbial profiles in other populations. From NCBI/SRA bioprojects, we retrieved the available salivary microbiome sequences from populations such as Bangladesh, Brazil, Japan, South Korea Germany, UK, and USA. Comparison of the salivary microbial profiles of the above-mentioned populations and the salivary microbiome composition in the samples collected from Qataris, revealed differences in the composition at both the phylum (Additional file 2: Figure S2A, B and data not shown) and genus levels in addition to microbial diversity (Additional file 2: Figure S2C), indicating a population-based variability in the salivary microbial profiles [12, 46, 47]. *Bacteroidetes* was the predominant bacteria among Qatari and German populations with 72% and 33% of the total phyla composition respectively. On the other hand, UK (78.32%), Brazil (50.25%), USA (40.62%), Bangladesh (32.11%), Japan (42.48%) and South Korean (39.91%) populations showed higher abundance of *Firmicutes* (Additional file 2: Figure S2B). Beta diversity measures using Bray–Curtis distances showed a significant clustering between different populations and this result was confirmed using Anosim test with a *P* value of 0.001 (Additional file 2: Figure S2C).

### Gender and the salivary microbiome

**Fig. 1:**
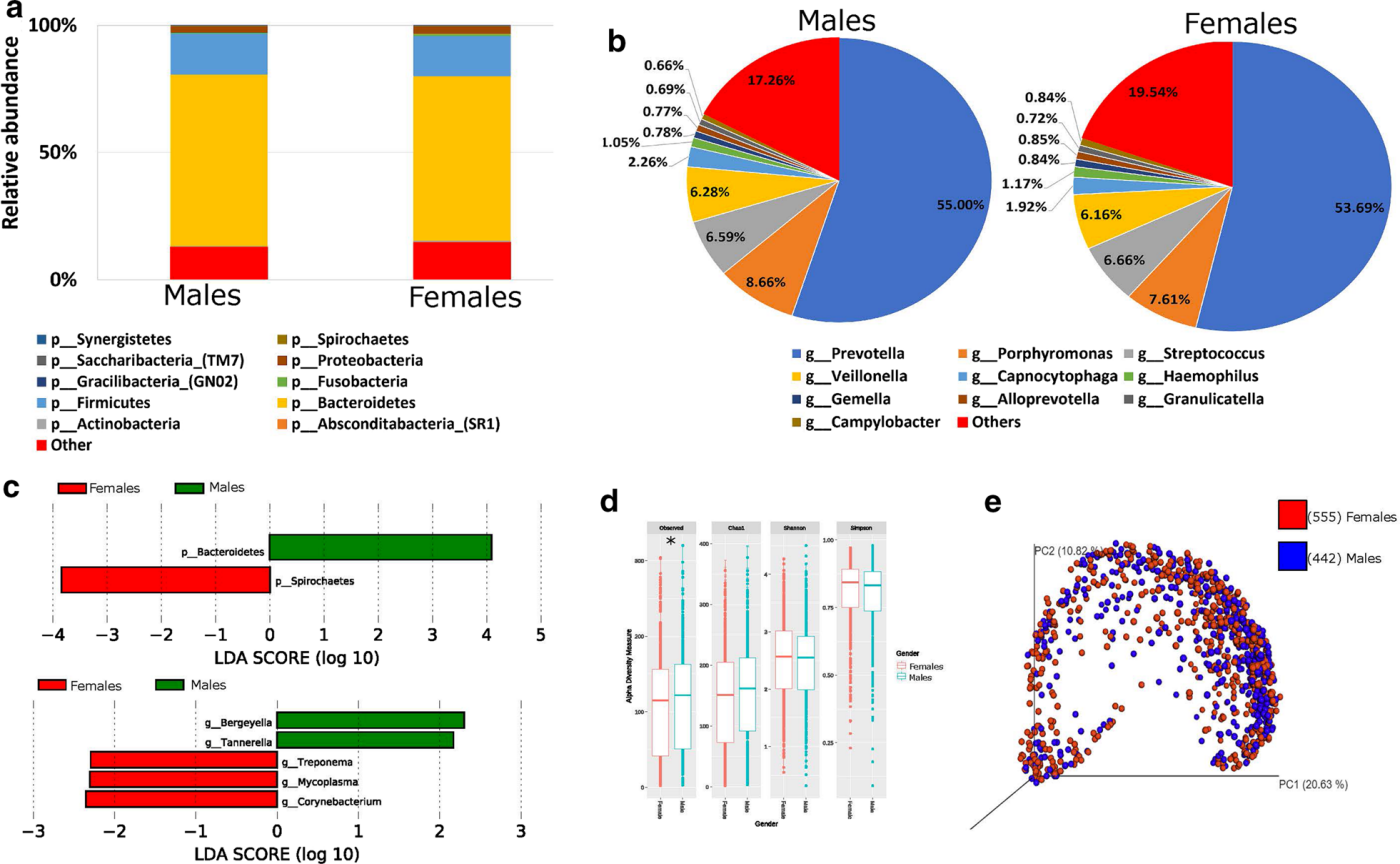
Gender and the salivary microbiome. Y-axis shows % of relative abundance; X-axis indicates the microbial abundance in males and females; each taxonomic category is shown by a different color: **a** at the phylum level; **b** at the genus level; **c** graphs of linear discriminant analysis (LDA) scores for differentially abundant bacterial phyla and genera; among the two groups. LDA scores indicate overrepresented bacteria in males (green) and females (red). Features with LDA scores ≥ 2 are presented. **d** Alpha diversity measures for the two groups. Alpha diversity was measured by the number of OTUs observed or by the Chao1, Shannon and Simpson diversity measures; **e** Principle Coordinates Analysis (PCoA) based on Bray–Curtis dissimilarities of salivary microbiome. Axes are scaled to the amount of variation explained; *P < 0.05

We assessed whether specific differences in the bacterial taxa were observed between males and females. The relative abundance of the salivary microbiome at the phylum level showed that *Bacteroidetes*, *Firmicutes*, *Proteobacteria* and *Fusobacterium* were the most common phyla observed in the saliva samples (Fig. 1a). A significant increase of *Bacteroidetes* was observed in males (67.3%) as compared to females (64.8%) (Fig. 1a, c—upper panel). At the genus level, *Prevotella*, *Porphyromonas*, *Streptococcus* and *Veillonella* were the top abundant members of the salivary microbiome observed in both males and females. *Bergeyella*, *Tannerella* genera were significantly higher in males while *Treponema*, *Mycoplasma* and *Corynebacterium* genera were significant higher in females (Fig. 1b, c—lower panel). The indices Chao1, observed OTUs, Shannon index, and Simpson were used to examine alpha diversity (Fig. 1d). Bacterial community richness (Chao1) and diversity (Shannon and Simpson) of the microbiome showed no significant differences between males and females (Fig. 1d). On the other hand, the observed species richness index was significantly higher (*P *= 0.042) among Qatari males than females (Fig. 1d). Beta diversity measures using Bray–Curtis distances did not show any group specific clustering, this result was also confirmed using Anosim test (Fig. 1e).

### The salivary microbiome composition and aging

**Fig. 2:**
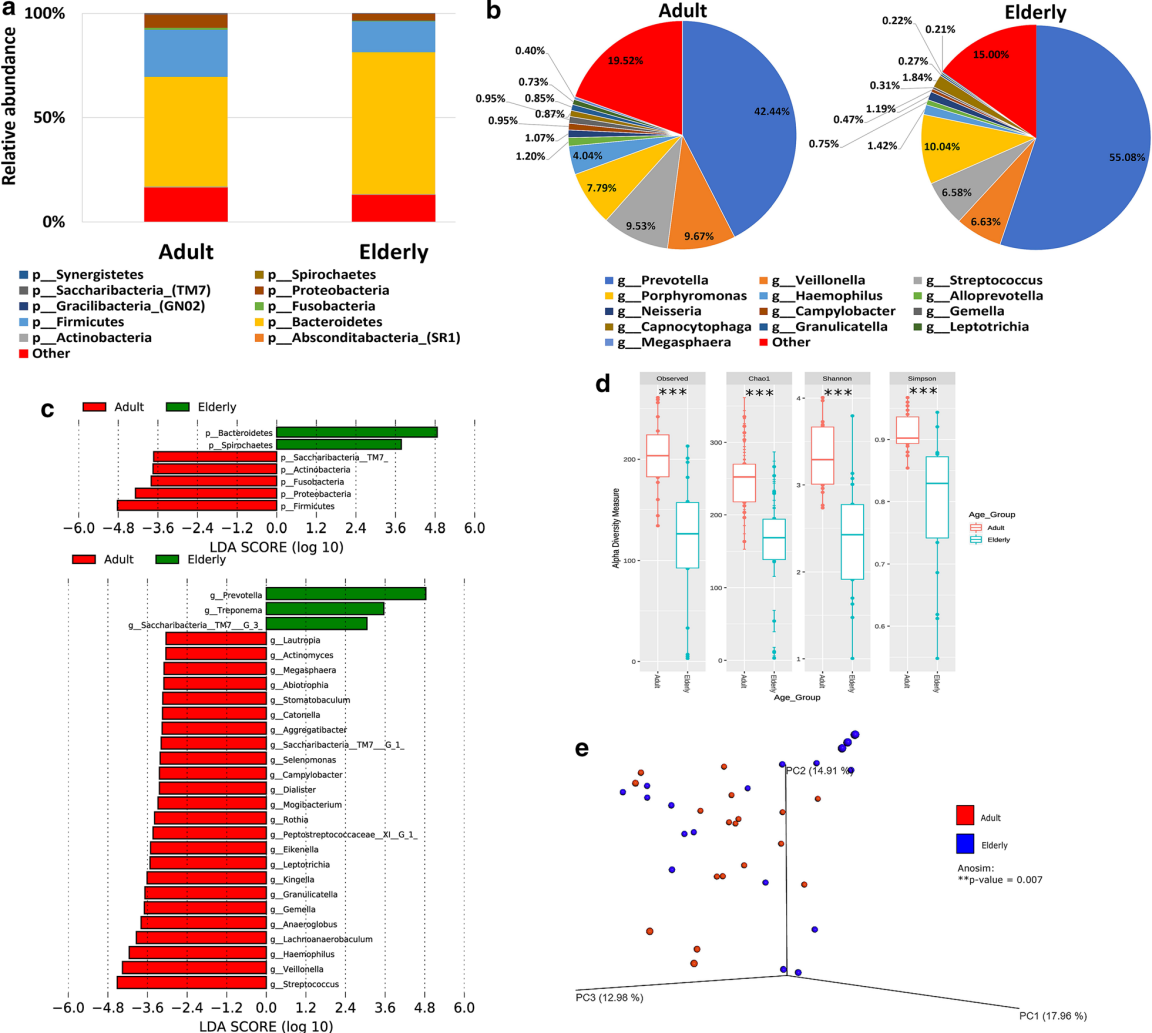
The salivary microbiome composition and aging. Y-axis shows % of relative abundance; X-axis indicates the microbial abundance in males and females; each taxonomic category is shown by a different color: **a** at the phylum level; **b** at the genus level; **c** graphs of linear discriminant analysis (LDA) scores for differentially abundant bacterial phyla and genera; among the two groups. LDA scores indicate overrepresented bacteria in Elderly (green) and Adults (red). Features with LDA scores ≥ 2 are presented. **d** Alpha diversity measures for the two groups. Alpha diversity was measured by the number of OTUs observed or by the Chao1, Shannon and Simpson diversity measures; **e** Principle Coordinates Analysis (PCoA) based on Bray–Curtis dissimilarities of salivary microbiome. Axes are scaled to the amount of variation explained; ***P < 0.001

Analysis of the relative abundance of the salivary microbiome in older participants (defined as people older than 65 years of age) revealed that *Bacteroidetes* and *Spirochaetes* were significantly higher in the elderly participants as compared to their adults’ counterparts (less than 65 years old) (Fig. 2a, c—upper panel). On the other hand, *Actinobacteria*, *Firmicutes*, *Fusobacteria*, *Proteobacteria* and *Saccharibacteria* were significantly higher in the adults (Fig. 2a, c—upper panel) compared to the older participants at the phylum level. Moreover, analysis at the genus level showed that the abundance of *Prevotella* and *Treponema* genera were significantly higher in the elderly participants, whereas, *Veillonella*, *Streptococcus*, *Mogibacterium*, *Megasphaera*, *Rothia* and *Camphylobacter* genera among others were significantly elevated in the adults participants (Fig. 2b, c—lower panel). Alpha-diversity measures indicated that the salivary microbiome in the elderly participants has a lower bacterial richness and diversity as compared to the adults (P value < 0.001) (Fig. 2d). In Bray–Curtis based beta diversity, although the clustering of the adults’ salivary microbiome was not conclusive; the anosim analysis of distance matrices revealed that there is a significant difference among the adults and elderly participants with a *P* value of 0.007 (Fig. 2e).

### The salivary microbiome composition and oral health

The study participants were stratified according to their answers recorded in the oral health section of the QBB baseline questionnaire. Those questions included whether the participant suffers from a bleeding gum, a mouth ulcer, painful gum, loose teeth, and where they have a denture or not (Table 1). Participants who answered No to all of the oral health-related questions were used as controls.

#### Bleeding gum and the salivary microbiome composition

**Fig. 3:**
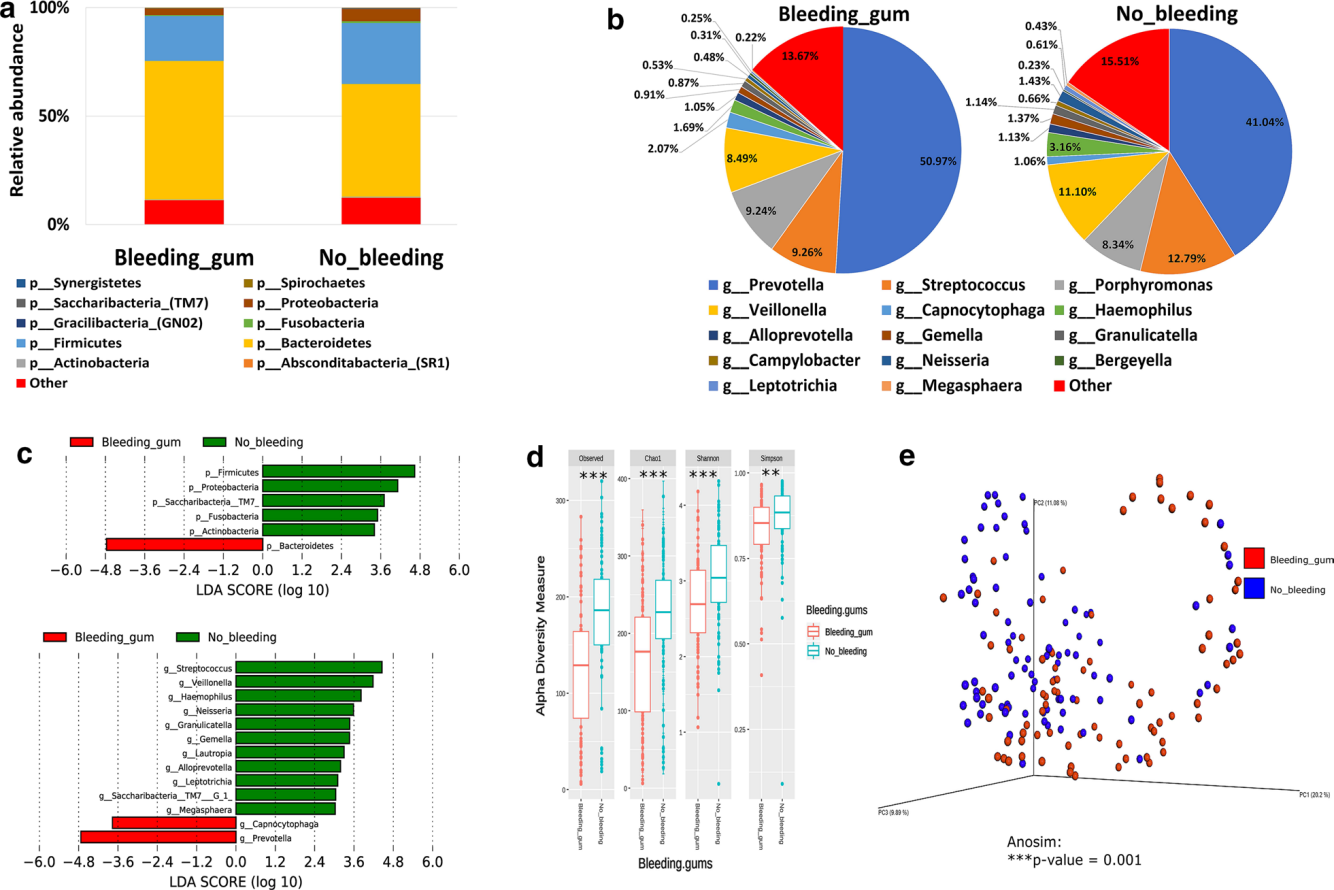
Bleeding gum and the salivary microbiome composition. Y-axis shows % of relative abundance; X-axis indicates the microbial abundance in males and females; each taxonomic category is shown by a different color: **a** at the phylum level; **b** at the genus level; **c** graphs of linear discriminant analysis (LDA) scores for differentially abundant bacterial phyla and genera; among the two groups. LDA scores indicate overrepresented bacteria in individuals that did not report bleeding (green) and the participants that reported bleeding gums (red). Features with LDA scores ≥ 2 are presented. **d** Alpha diversity measures for the two groups. Alpha diversity was measured by the number of OTUs observed or by the Chao1, Shannon and Simpson diversity measures; **e** Principle Coordinates Analysis (PCoA) based on Bray–Curtis dissimilarities of salivary microbiome. Axes are scaled to the amount of variation explained; **P < 0.01, ***P < 0.001

A total of 84 participants reported suffering from a bleeding gum (Table 1). The salivary microbiome composition in those participants was compared to age and gender matching controls. Our analysis revealed that participants who suffer from bleeding gum have significantly higher *Bacteroidetes* at the phylum level and more *Prevotella* at the genus level (Fig. 3a, c—upper panel). On the other hand, a significant increase in *Actinobacteria*, *Firmicutes*, *Proteobacteria* and *Saccharibacteria* phyla was observed in the control participants. *Streptococcus*, *Veillonella*, *Haemophilus*, *Granulicatella* and *Lautrophia* genera among others were significantly more abundant in the control group (Fig. 3b, c—lower panel). All indices of alpha diversity indicated that the salivary microbiome in the participants suffering from a bleeding gum is less diverse compared to the controls (**P < 0.01, ***P < 0.001) (Fig. 3d). In the Bray–Curtis based beta diversity, the anosim analysis of distance matrices revealed that there is a significant difference among the two groups with a *P* value of 0.001 (Fig. 3e). Similar results were also observed in participants that reported having painful gum (a total of 81 participants) or loose teeth (a total of 38 participants) (Additional file 3: Figure S3 and Additional file 4: Figure S4).

#### Mouth ulceration and the salivary microbiome composition

**Fig. 4:**
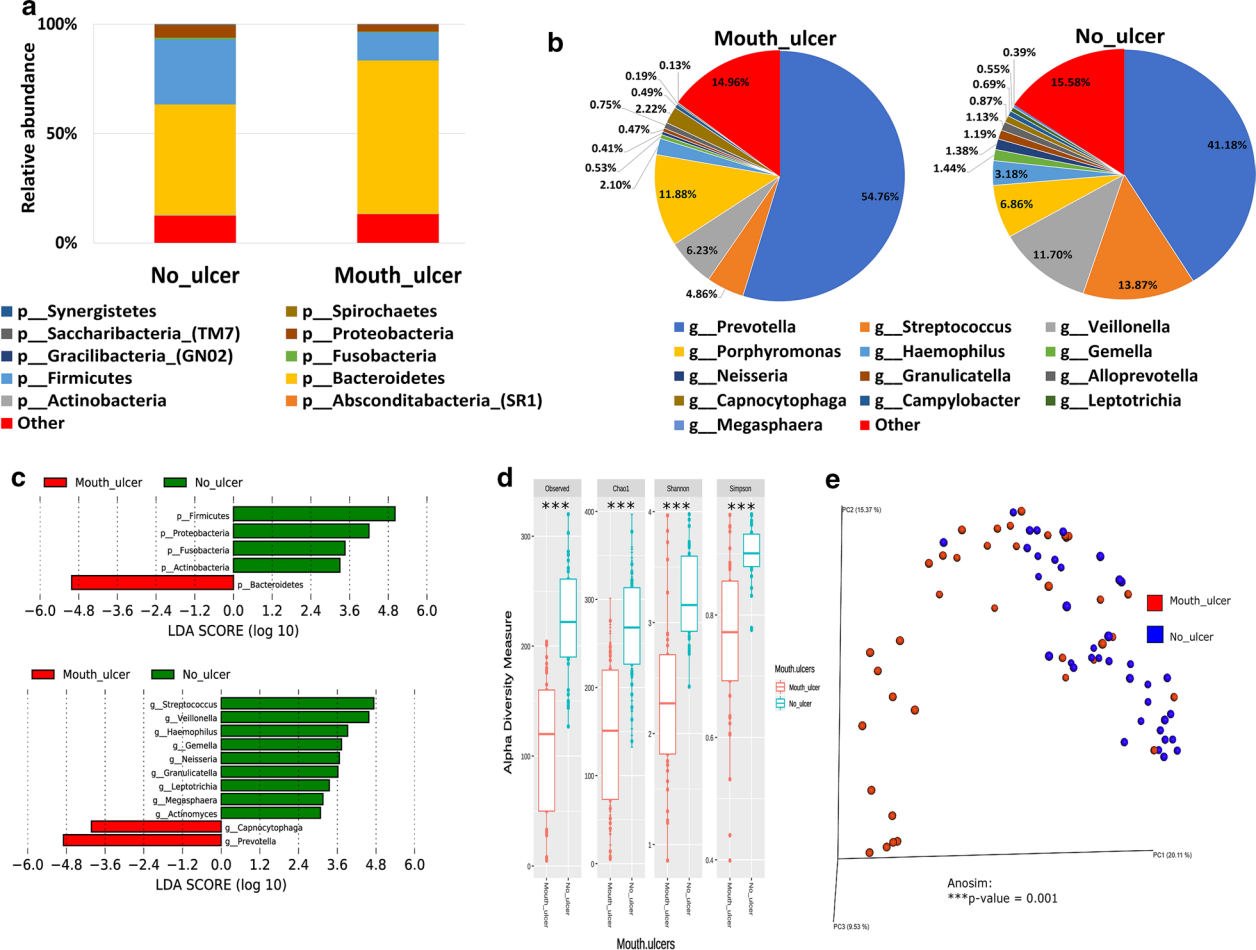
Mouth ulceration and the salivary microbiome composition. Y-axis shows % of relative abundance; X-axis indicates the microbial abundance in males and females; each taxonomic category is shown by a different color: **a** at the phylum level; **b** at the genus level; **c** graphs of linear discriminant analysis (LDA) scores for differentially abundant bacterial phyla and genera; among the two groups. LDA scores indicate overrepresented bacteria in individuals that did not report any mouth ulcer (green) and the participants that reported having mouth ulcers (red). Features with LDA scores ≥ 2 are presented. **d** Alpha diversity measures for the two groups. Alpha diversity was measured by the number of OTUs observed or by the Chao1, Shannon and Simpson diversity measures; **e** Principle Coordinates Analysis (PCoA) based on Bray–Curtis dissimilarities of salivary microbiome. Axes are scaled to the amount of variation explained; ***P < 0.001

A total of 37 participants reported suffering from mouth ulcers (Table 1). The salivary microbiome composition in those participants was compared to age and gender-matching controls. Our analysis revealed that *Bacteroidetes* were significantly higher at the phyla level in participants suffering from mouth ulcers (Fig. 4a, c—upper panel), and significantly more *Prevotella* and *Capnocytophaga* were observed at the genus level (Fig. 4b, c—lower panel). Similar to the group of participants suffering from bleeding gums, our data show that *Actinobacteria*, *Firmicutes*, *Proteobacteria* and *Saccharibacteria* at the phyla level and *Streptococcus*, *Veillonella*, *Haemophilus*, *Gemella*, *Granulicatella*, *Megasphaera* and *Leptotrichia* genera were significantly abundant in the control group (Fig. 4c—lower panel). All indices of alpha diversity indicated that the salivary microbiome in the participants suffering from mouth ulcers is less diverse compared to the controls (***P < 0.001) (Fig. 4d). In the Bray–Curtis based beta diversity, the anosim analysis of distance matrices revealed that there is a significant difference among the two groups with a *P* value of 0.001 (Fig. 4e).

#### Denture use and the salivary microbiome composition

**Fig. 5:**
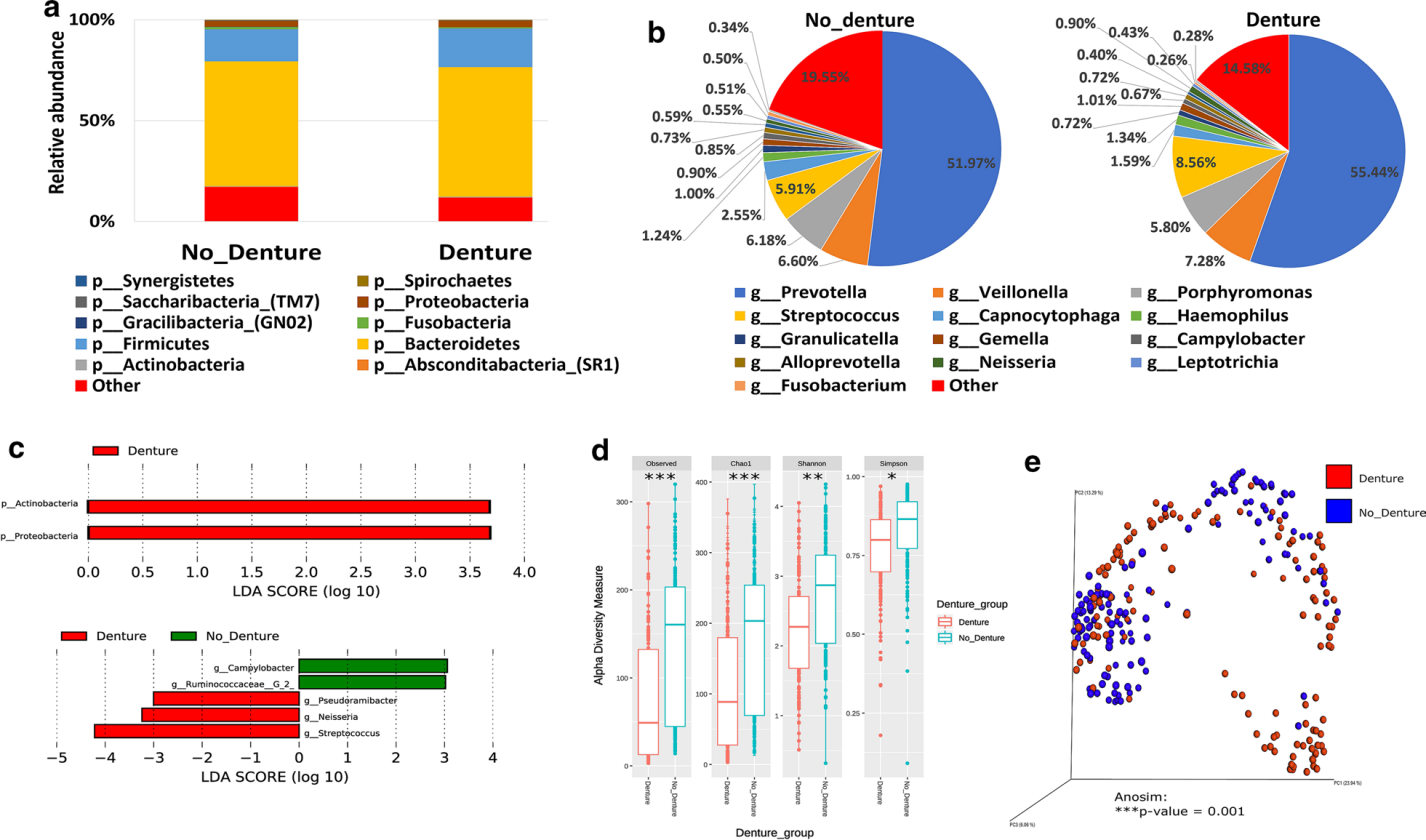
Denture use and the salivary microbiome composition. Y-axis shows % of relative abundance; X-axis indicates the microbial abundance in males and females; each taxonomic category is shown by a different color: **a** at the phylum level; **b** at the genus level; **c** graphs of linear discriminant analysis (LDA) scores for differentially abundant bacterial phyla and genera; among the two groups. LDA scores indicate overrepresented bacteria in individuals that did not use dentures (green) and the participants that reported using dentures (red). Features with LDA scores ≥ 2 are presented. **d** Alpha diversity measures for the two groups. Alpha diversity was measured by the number of OTUs observed or by the Chao1, Shannon and Simpson diversity measures; **e** Principle Coordinates Analysis (PCoA) based on Bray–Curtis dissimilarities of salivary microbiome. Axes are scaled to the amount of variation explained; *P < 0.05, **P < 0.01, ***P < 0.001

A total of 136 participants reported using dentures (Table 1). The salivary microbiome composition in those participants was compared to age and gender-matching controls. Our analysis revealed that *Proteobacteria* and *Actinobacteria* were significantly higher at the phylum level in participants using dentures (Fig. 5a, c—upper panel), whereas significantly more *Streptococcus*, *Neisseria* and *Pseudoramibacter* were observed at the genus level (Fig. 5b, c—lower panel). Moreover, our data show that *Campylobacter* and *Ruminococcaceae* genera were significantly higher in the control group (Fig. 5c—lower panel). All indices of alpha diversity indicated that the salivary microbiome in the participants using dentures is less diverse compared to the controls (*P < 0.05, **P < 0.01, ***P < 0.001) (Fig. 5d). In the Bray–Curtis based beta diversity, the anosim analysis of distance matrices revealed that there is a significant difference among the two groups with a *P* value of 0.001 (Fig. 5e).

### The salivary microbiome composition is influenced by smoking habits

**Fig. 6:**
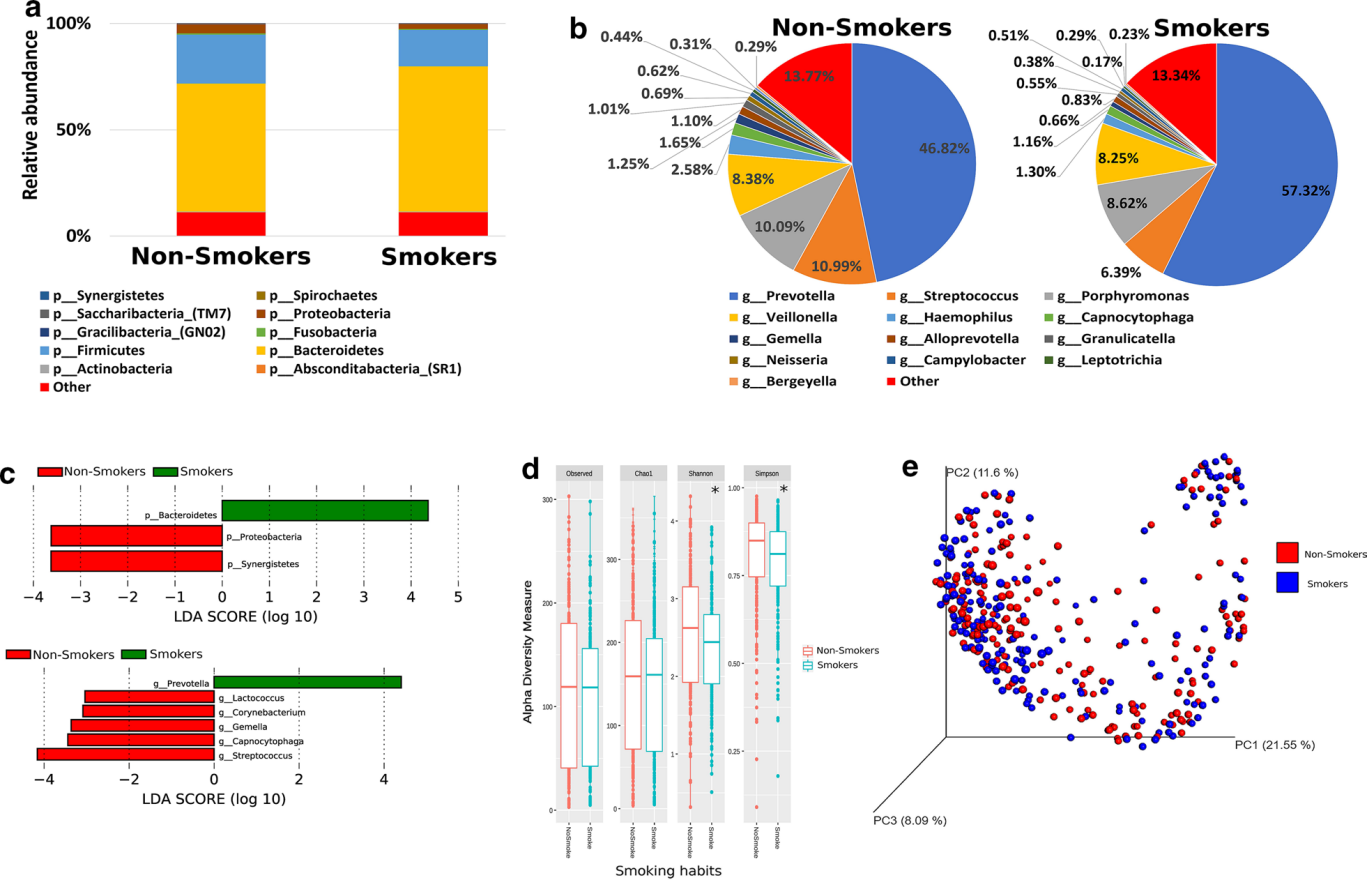
The salivary microbiome composition is influenced by smoking habits. Y-axis shows % of relative abundance; X-axis indicates the microbial abundance in males and females; each taxonomic category is shown by a different color: **a** at the phylum level; **b** at the genus level; **c** graphs of linear discriminant analysis (LDA) scores for differentially abundant bacterial phyla and genera; among the two groups. LDA scores indicate overrepresented bacteria in smokers (green) and non- smokers (red). Features with LDA scores ≥ 2 are presented. **d** Alpha diversity measures for the two groups. Alpha diversity was measured by the number of OTUs observed or by the Chao1, Shannon and Simpson diversity measures; **e** Principle Coordinates Analysis (PCoA) based on Bray–Curtis dissimilarities of salivary microbiome. Axes are scaled to the amount of variation explained; *P < 0.05

Based on their smoking habits, participants were classified into smokers (264 participants) and non-smokers (733 participants). By comparing the two groups, our analysis revealed that *Bacteroidetes* at the phylum level and *Prevotella* genus were significantly higher in the smokers’ group (Fig. 6a–c). On the other hand, *Proteobacteria* and *Synergistetes* at the phylum level, *Lactococcus*, *Corynebacterium*, *Gemella*, *Capnocytophaga* and *Streptococcus* at the genus level were significantly higher in the non-smokers (Fig. 6a–c). The alpha diversity measures indicated that the salivary microbiome of the non-smokers is significantly more diverse as compared to the smokers (*P < 0.05), but there was no significant difference observed in species richness between the two groups (Fig. 6d). On the other hand, beta diversity measures did not show any significant clustering or distance difference between the two groups (Fig. 6e).

### Influence of coffee and tea consumption on the salivary microbiome composition

The frequency of coffee and tea intake including Arabic coffee, instant coffee, filtered coffee, cappuccino, red tea, herbal tea and Karak (special type of tea mixed with milk, usually offered in the Arabian Gulf countries) were assessed using the dietary questionnaire. From the cohort, the participants were further derived into two groups of coffee/tea drinkers (consuming more than 1–3 cups per week) and non-drinkers (consuming less than 1–3 cups of tea/coffee per month). Among the participants, 110 were coffee drinkers, 887 were non-coffee drinkers, 229 participants were tea drinkers and 768 were non-tea drinkers. The salivary microbiome composition was analyzed in the coffee/tea drinkers and non-drinkers in order to assess the effect of tea and coffee consumption on the salivary microbiome composition.

#### Coffee consumption

**Fig. 7:**
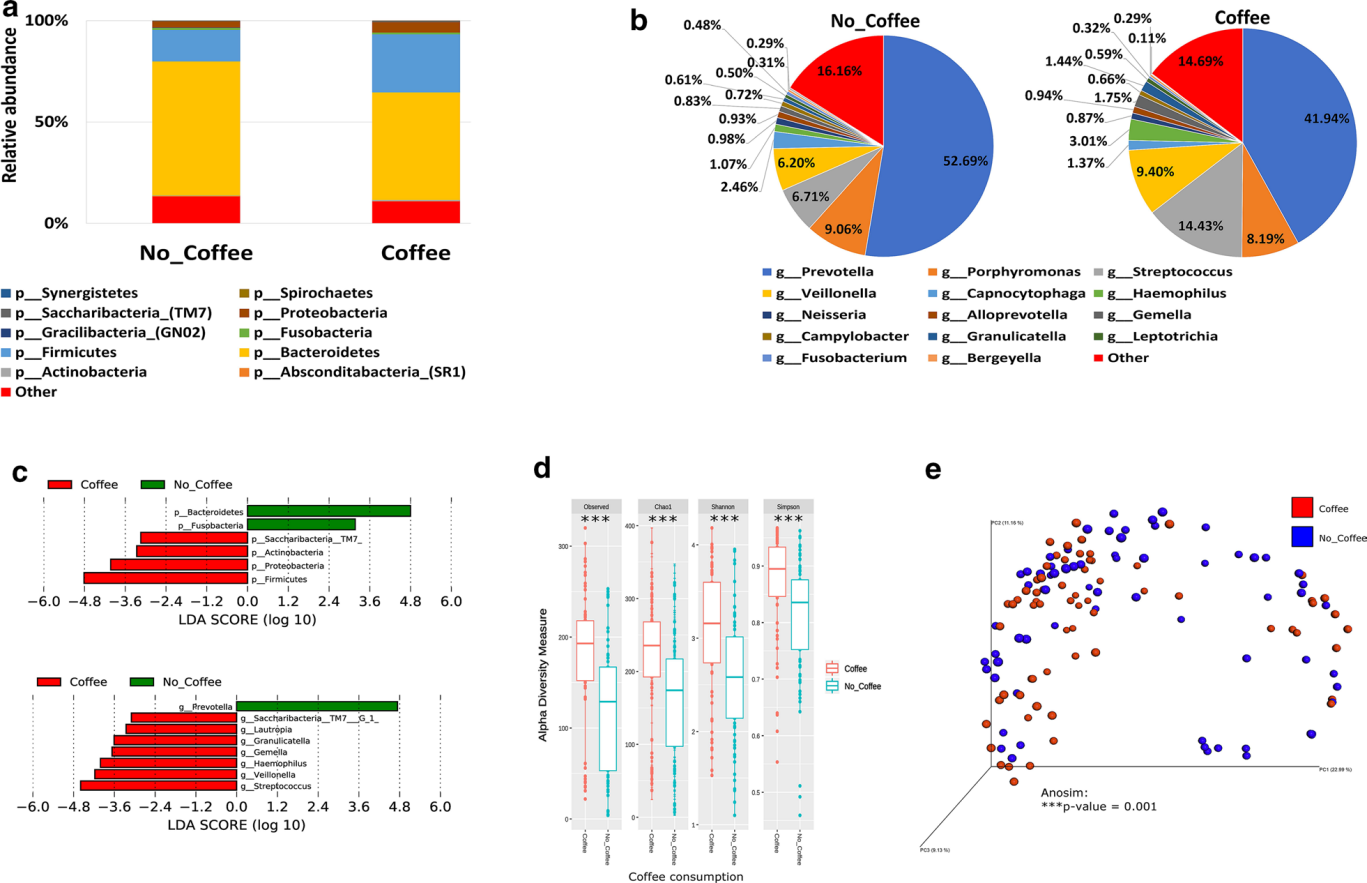
Influence of Coffee consumption on the salivary microbiome composition. Y-axis shows % of relative abundance; X-axis indicates the microbial abundance in males and females; each taxonomic category is shown by a different color: **a** at the phylum level; **b** at the genus level; **c** graphs of linear discriminant analysis (LDA) scores for differentially abundant bacterial phyla and genera; among the two groups. LDA scores indicate overrepresented bacteria in individuals that do not drink coffee (green) and the participants that are considered coffee drinkers (red). Features with LDA scores ≥ 2 are presented. **d** Alpha diversity measures for the two groups. Alpha diversity was measured by the number of OTUs observed or by the Chao1, Shannon and Simpson diversity measures; **e** Principle Coordinates Analysis (PCoA) based on Bray–Curtis dissimilarities of salivary microbiome. Axes are scaled to the amount of variation explained; ***P < 0.001

Our data shows that *Actinobacteria*, *Firmicutes*, *Proteobacteria* and *Saccharibacteria* phyla were significantly higher in the saliva samples collected from coffee drinkers as compared to the non-coffee drinkers, whereas, *Bacteroidetes* and *Fusobacteria* were the significant phyla in the non-coffee drinkers (Fig. 7a, c—upper panel). At the genus level, *Streptococcus*, *Veillonella*, *Haemophilus*, *Gemella*, *Granulicatella* and *Lautrophia* were significantly abundant in the saliva samples collected from coffee drinkers while *Prevotella* was the most significantly abundant genus observed in the non-coffee drinkers (Fig. 7b, c—lower panel). All indices of alpha diversity indicated that the salivary microbiome in the non-coffee drinkers is less diverse compared to the coffee drinkers (***P < 0.001) (Fig. 7d). In the Bray–Curtis based beta diversity measure, the anosim analysis of distance matrices revealed that there is a significant difference among the two groups with a *P* value of 0.001 (Fig. 7e).

#### Tea consumption

**Fig. 8:**
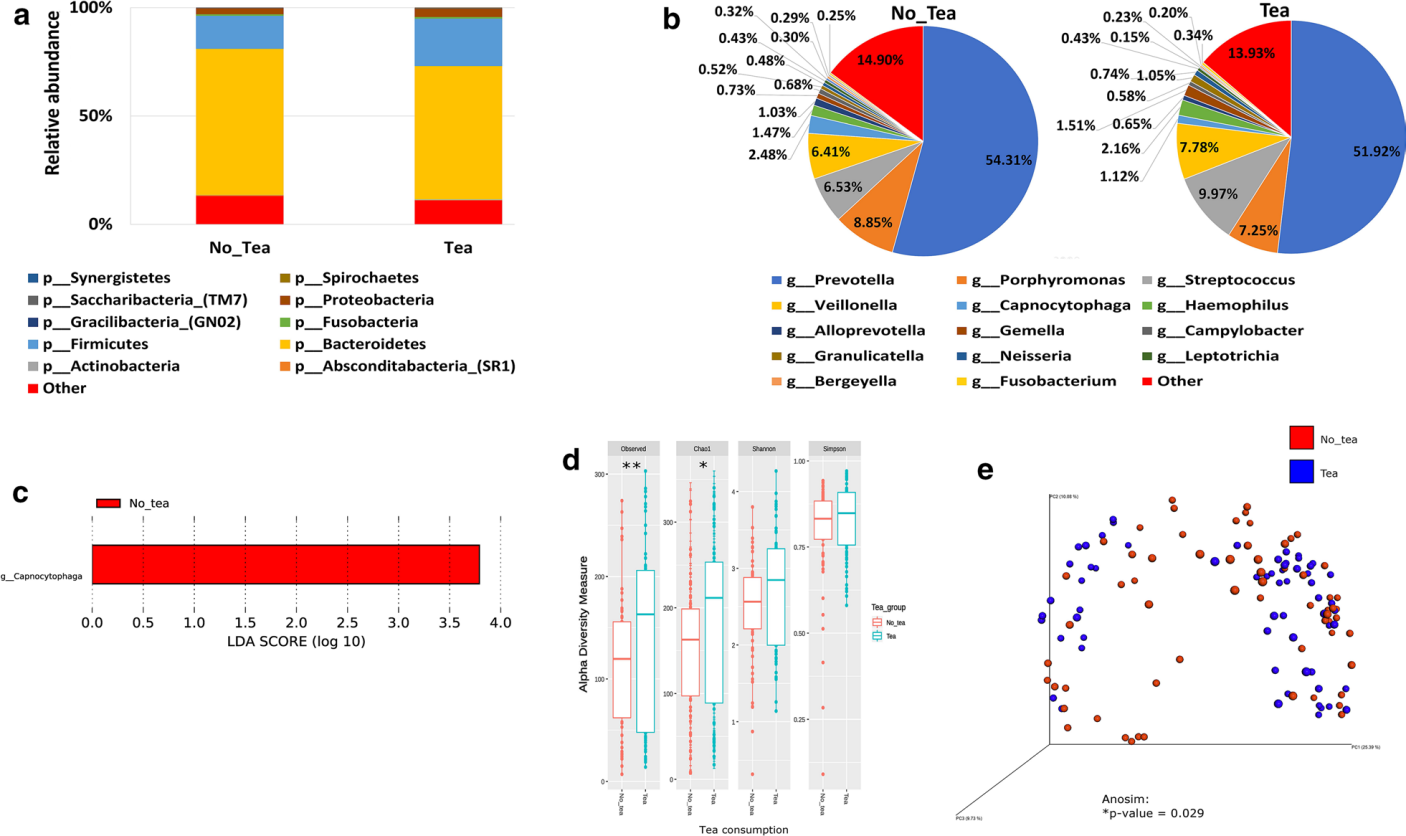
Influence of tea consumption on the salivary microbiome composition. Y-axis shows % of relative abundance; X-axis indicates the microbial abundance in males and females; each taxonomic category is shown by a different color: **a** at the phylum level; **b** at the genus level; **c** Graphs of linear discriminant analysis (LDA) scores for differentially abundant bacterial phyla and genera; among the two groups. Features with LDA scores ≥ 2 are presented. **d** Alpha diversity measures for the two groups. Alpha diversity was measured by the number of OTUs observed or by the Chao1, Shannon and Simpson diversity measures; **e** Principle Coordinates Analysis (PCoA) based on Bray–Curtis dissimilarities of salivary microbiome. Axes are scaled to the amount of variation explained; *P < 0.05, **P < 0.01

Our data shows that although the abundance of *Actinobacteria*, *Bacteroidetes*, *Firmicutes* and *Proteobacteria* was higher in the coffee drinkers (Fig. 7a, c—upper panel), no significant difference was observed in the tea drinkers (Fig. 8a, c). At the genus level *Capnocytophaga* was significantly higher in the non- tea drinkers’ group in comparison with the tea drinkers (Fig. 8b, c). The alpha diversity measures showed that a significant increase in the microbial richness was observed in the tea drinking group, (*P < 0.05, **P < 0.01) but no changes were observed in the microbial diversity indices (Fig. 8d). In the Bray–Curtis based beta diversity measure, the anosim analysis of distance matrices revealed that there is a significant difference among the two groups with a *P* value of 0.029 (Fig. 8e).

## Discussion

The purpose of this study was to examine the salivary microbiome composition in the Qatari population, and assess its association with gender, age, oral health, smoking coffee and tea consumption. We characterized the salivary microbiome of 997 Qatari participants including 442 males and 555 females. To the best of our knowledge, this is one of the largest population-based studies assessing the salivary microbiome and is the first study to characterize the salivary microbiome in an Arab population like Qataris.

We show that the bacterial profile, in spite of its high diversity, was dominated by the phylum *Bacteroidetes*, which is different compared to other populations like Bangladesh, UK, Japan, South Korea and Brazil, where *Firmicutes* was the most predominant phylum. In this study, we show that *Prevotella*, *Porphyromonas*, *Streptococcus*, *Veillonella*, *Haemophilus*, *Gemella*, and *Neisseria* were the most common members of the Qatari salivary microbiome, with *Prevotella* being the most predominant genus. In contrast, *Streptococcus* is the most abundant genus in the UK, South Korea, Japan and US populations [46, 48–53]. The differences observed in the Qatari salivary microbiome compared to other populations, may be influenced by various factors, including host genetics, diet and environmental factors [49].

In this study, we show that the observed species richness index was significantly higher among Qatari males than females, with a significant shift in particular genera like *Bergeyella*, *Tannerella* in males and *Treponema*, *Mycoplasma* and *Corynebacterium* in females. This may be influenced by many factors including hormones and body mass index as previously reported [50, 54–56].

Our data show that the salivary microbial composition is associated with age, which is in accordance to previously reported results [57]. The senior participants included in our cohort showed a reduced bacterial diversity and significantly increased *Prevotella*, when compared to the younger adults. Proteolytic bacteria such as *Prevotella* are known to degrade proteins and peptides and are associated with periodontitis [28, 58]. It is known that the saliva composition changes with age due to the slower salivary flow [59], inadequate oral care, increase of several inflammatory mediators [60], systemic diseases and other additional environmental factors that together will affect the microbial composition in the saliva [28, 61]. The adult salivary microbial population this cohort was more diverse including microbes like *Streptococcus, Haemophilus*, *Rothia and Veillonella and Lautrophia*, some are known to degrade carbohydrates [50].

We also show that participants that suffer from various oral conditions including bleeding or painful gum, loose teeth or mouth ulcers have lower salivary microbial diversity; with *Prevotella* being the most predominant member. Previous studies have shown an association between increased abundance of *Prevotella*, aphthous ulcers and periodontal disease (more specifically gingivitis) [62, 63]. Our data indicates that the alpha diversity of the salivary microbiome in the control groups was significantly higher in comparison to those suffering from poor oral health, which is similar to previously reported results [64]. Our data show that usage of denture is positively correlated with age and is closely linked with the oral health status [65]. In this study, we show that using denture reduces the diversity of the salivary microbiome with an enrichment of both *Proteobacteria* and *Actinobacteria* phyla while *Streptococcus* and *Neisseria* were enriched at the genus levels. This result is supported by other studies comparing biofilms forming on natural teeth against those forming on denture teeth [66, 67].

Smoking modulates the microbial composition of various body sites including upper gut, respiratory tract and the oral microbiome [68–71]. Prior studies had shown that smoking disrupts the microbial homeostasis leading to various oral disease such as gingivitis and dental loss [72]. Our analysis revealed that smoking reduced the salivary microbial diversity and *Bacteroidetes* was the most abundant phylum observed, which has been reported previously [31]. Moreover, the genus *Prevotella* was more abundant in the smokers compared to non-smokers, suggesting therefore an increased vulnerability of the smokers to develop oral diseases such as gingivitis [31]. This suggests that smoking has to always be considered in the future when assessing the oral microbiome composition, as it clearly affects the salivary microbiome composition.

Coffee and tea are commonly consumed beverages in most populations, and both were heavily studied to assess their health benefits [73–75]. In our cohort, around 11% of the Qatari participants reported drinking coffee and 23% reported drinking tea. While some papers reported that both coffee and tea affect the microbial composition of the saliva [73, 76, 77]; our data show that a significant increase in *Granulicatella*, *Gemella*, *Streptococcus* and *Lautrophia* along with an increased microbial richness and diversity is observed in the coffee but not in the tea drinkers. Higher abundance of *Granulicatella* in the saliva of coffee drinkers was previously reported [73].

Our data showed that *Prevotella* is the most abundant bacteria observed in the salivary microbiome of the Qatari adult population. *Prevotella* is one of the commonly reported members of the oral microbiome [78] and it has been linked previously to various inflammatory conditions such as rheumatoid arthritis, metabolic disorders and periodontal infections among others [79]. Based on gender, we did not observe any significant difference in the abundance of *Prevotella* between males (55%) and females (53.69%) in this cohort. We show that, *Prevotella* was more abundant in subjects with mouth ulcers (54.76%), bleeding gum (50.97%) as compared to the healthy individuals (41.04%, 41.10%). *Prevotella* is higher in the group of smokers (57.32%) as compared to non-smokers (46.82%). Various *Prevotella* species can play different roles in health and disease [79–81]. In a recent study of 161 healthy Italian participants, the salivary microbiome was classified into *Prevotella*-dominant type, *Streptococcus*/*Gemella*-dominant type and *Neisseria*/*Fusobacterium*-dominant types [82]. The microbial co-occurrence/exclusion pattern was explained by the microorganisms need to nutrients that can be provided by a selective group of bacteria [83, 84]. Our study divulges the decrease in Co-occurring *Prevotella*/*Porphyromonas* shifts in healthy controls and increase in diseased cases. In another study *Prevotella histicola* was shown to have a boosting effect of Copaxone, used to treat patients with multiple sclerosis [85]. Most of the members of *Prevotella* remain to be considered as commensals in healthy participants, which then turn to pathogens in oral infections and immunocompromised patients. *Prevotella* is a beneficial microbe that is associated with plant-rich diet and the diverse *Prevotella* species will have differences in responding to the diet and health status of hosts [86]. More research is needed in order to look further deeper into *Prevotella*’s potential and its interactions with its host and other bacteria for therapeutic use in clinical practice.

## Conclusions

In summary, this large-scale population-based study described for the first time the salivary microbiome profiles in the Qatari population. Our data indicated population-specific microbial composition, with major phyla differences between Qatari population and populations from Brazil, Japan, South Korea, Germany, UK and USA [12, 46, 47]. We also show that the salivary microbiome composition is associated with gender, age, oral health, smoking and coffee intake. The scope of this study is mainly to give a picture of the salivary microbiome and their changes related to various factors. However, due to the limitation of using 16S rRNA gene sequencing, we cannot conclude on the function of those microbial changes in relation to various conditions. Future work on the microbial transcriptomics and metabolomics is needed to provide deeper insights into the role of the salivary microbiome in health and disease.

## Materials and methods

### Ethics statement

The study was approved by the Institutional Review board (IRB) of Sidra Medicine under (protocol #1510001907) and by Qatar Biobank (QBB) under protocol (E/2017/RES-ACC-0046/0003). All study participants signed an informed consent prior to sample collection. All experiments were performed in accordance with the approved guidelines.

### Clinical data

In this project, an agreement between QBB and Sidra Medicine was signed in order to collect de-identified salivary samples, phenotypic and clinical data from a total of 997 Qatari participants that were selected randomly. All participants were 18 years old and above. No exclusion criteria were applied in this studied cohort. The cohort consisted of 442 males and 555 females (Table 1). All participants answered the baseline questionnaire to describe their oral hygiene, smoking and dietary habits.

### Sample collection

Saliva samples were collected in QBB according to a standard technique. A total of 5 mL of spontaneous, whole, unstimulated saliva was collected into a 50 mL sterile DNA-free Falcon tube from each participant. The samples were divided into 0.4 mL aliquots and stored at − 80 °C until further analysis. The aliquots were received from QBB for total salivary DNA extraction.

### DNA extraction and 16S rRNA gene sequencing

The total salivary DNA were extracted using automated QIAsymphony protocol (Qiagen, Hilden, Germany) following the manufacturer’s instructions. DNA purity was evaluated by the A260/A280 ratio using a NanoDrop 7000 Spectrophotometer (Thermo Fisher Scientific, Waltham, MA, USA), and the DNA integrity was checked on a 1% agarose by gel electrophoresis.

The V1–V3 regions of the 16S rRNA gene were amplified using various forward primers: 27F with 12 bp golay barcodes containing a specific Illumina 5′ adapter for each sample and a common reverse primer 515 R [87, 88]. In brief, PCR was performed in triplicate in a 50 μL reaction mixture containing 10 ng of template DNA and 2x Phusion HotStart Ready Mix (Thermo Scientific™). The following thermal cycling conditions were used: 5 min of initial denaturation at 94 °C; 25 cycles of denaturation at 94 °C for 30 s, annealing at 62 °C for 30 s, extension at 72 °C for 30 s; and a final extension at 72 °C for 10 min. The amplified PCR products of approximately 650 bp in size from each sample were pooled in equimolar concentrations according to the manufacturer’s instructions (Illumina, Inc., San Diego, CA, USA). This pooled PCR product was purified using AgenCourt AMPure XP magnetic beads (Beckman Coulter). High throughput sequencing was performed on an Illumina MiSeq2×300 platform in accordance with the Manufacturer’s instructions. Image analysis and base calling were carried out directly on the MiSeq.

### Taxonomic classification of the salivary microbiome

Sequenced data were demultiplexed using MiSeq Control Software (MCS). Demultiplexed data were revised for quality control using FastQC [89]. Forward and reverse end sequences of respective samples were merged through PEAR tool [90] and sequence reads of quality score < 20 were discarded. All merged reads were trimmed to 160 bp > Reads < 500 bp using Trimmomatic tool [91]. Trimmed FASTQ files were converted into FASTA files. Demultiplexed FASTA files were analyzed using QIIME (Quantitative Insights Into Microbial Ecology) v1.9.0 pipeline [92]. Operational taxonomic units (OTUs) were generated by aligning against the Human Oral Microbiome Database (HOMD RefSeq, Version 15.1) with a confidence threshold of 97% [93]. For the comparison of salivary microbiome profiles, Sequence Read Archive (SRA) files from the bioprojects of other populations such as Brazil (PRJNA504439), Bangladesh (PRJEB23323), United States of America (USA) (PRJNA421776), South Korea (PRJDB2879), Germany (PRJNA387918), United Kingdom (UK) (PRJEB9010) and Japan (PRJDB4107) were retrieved. The Operational taxonomic units (OTUs) of different population were generated by aligning against Greengenes Database (Version:gg_13_8) with a confidence threshold of 97%.

### Significant abundances of the salivary microbiome and diversity analyses

Linear Discriminant Analysis Effect Size (LEfSe) [94] was used to find differentially abundant taxa between the studied categories, with per-sample normalization to 1 million, an alpha cut-off value of 0.05 for the Kruskal–Wallis factorial test, and a threshold for discriminative features at a logarithmic LDA score > 2. Alpha diversity was measured by R software, using the phyloseq package [95]. Beta diversity was represented using Phylogenetic beta diversity metrics [96] and non-phylogenetic beta diversity metrics and the differences in the beta diversity were presented as principle coordinate analysis using QIIME.

### Statistical analysis

Statistical significance of the alpha diversity measures such as Observed, Chao1, Shannon and Simpson indices were calculated using minitab 17 (Minitab statistical software). P-values lower than 0.05 were considered statistically significant. Analysis of similarities software (Anosim) was used to calculate the distance matrix difference between the categories included in this study (adult versus elderly, males versus females, etc.) using Bray–Curtis beta diversity parameters [92].

## Supplementary information

**Additional file 1: Figure S1.** The salivary microbiome of the Qatari population Relative abundance of the total cohort. Y-axis shows  % of relative abundance; X-axis indicates the abundance for Qatari population; each taxonomic category is shown by a different color A) phylum level B) genus level.

**Additional file 2: Figure S2.** The salivary microbiome of the other national population. A) Y-axis shows the percentage of relative abundance; X-axis reflects various populations included. Colors in the bar graph reflect bacterial phyla. B) Relative abundance table of the salivary microbiome in various populations at phylum level C) Principle Coordinates Analysis (PCoA) based on Bray–Curtis dissimilarities of the salivary microbiome. Axes are scaled to the amount of variation explained; ***P < 0.001.

**Additional file 3: Figure S3.** Salivary microbiome of Participants with Painful gum. Y-axis shows  % of relative abundance; X-axis indicates the abundance; each taxonomic category is shown by a different color a) phylum level b) genus level c) Graphs of linear discriminant analysis (LDA) scores for differentially abundant bacterial phyla and genera; among the groups. LDA scores indicate overrepresented bacteria in each group. Features with LDA scores ≥ 2 are presented. d) Alpha diversity measures were used to compare the two groups. Alpha diversity was measured by the number of OTUs observed, by the Chao1 index, in addition to Shannon and Simpson diversity measures, e) Principle Coordinates Analysis (PCoA) based on Bray–Curtis dissimilarities of the salivary microbiome. Axes are scaled to the amount of variation explained; ***P < 0.001.

**Additional file 4: Figure S4.** Salivary microbiome of participants with Loose teeth. Y-axis shows  % of relative abundance; X-axis indicates the abundance; each taxonomic category is shown by a different color a) phylum level b) genus level c) Graphs of linear discriminant analysis (LDA) scores for differentially abundant bacterial phyla and genera; among the groups. LDA scores indicate overrepresented bacteria in each group. Features with LDA scores ≥ 2 are presented. d) Alpha diversity measures were used to compare the two groups. Alpha diversity was measured by the number of OTUs observed, by the Chao1 index, Shannon and Simpson diversity measures, e) Principle Coordinates Analysis (PCoA) based on Bray–Curtis dissimilarities of salivary microbiome. Axes are scaled to the amount of variation explained; **P < 0.01, ***P < 0.001.

## Data Availability

The datasets used and/or analyzed during the current study are available upon request and after approval of QBB.
