# Supplementary figures and images for: Profiling the Salivary microbiome of the Qatari population
Source: J Transl Med. 2020 Mar 14;18:127. doi: 10.1186/s12967-020-02291-2 (PMC7071716; doi:10.1186/s12967-020-02291-2)

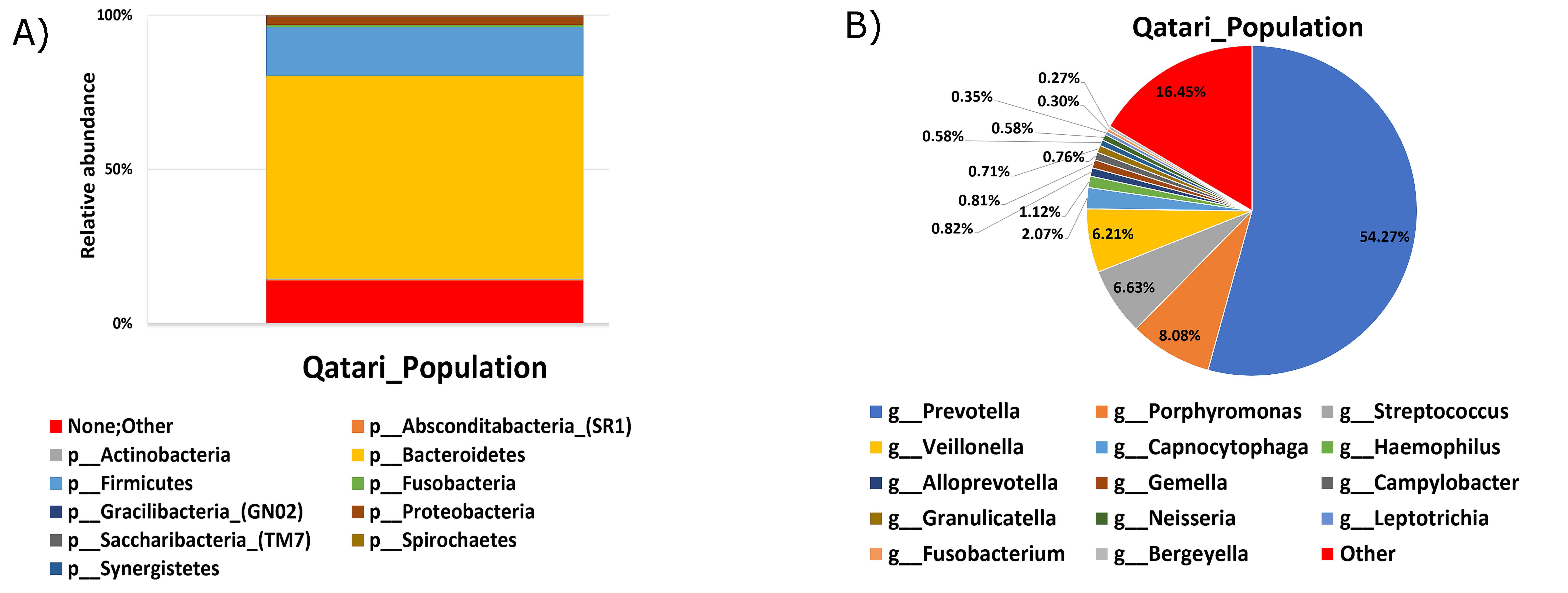

Supplement: Supplementary file 1 — Additional file 1: Figure S1. The salivary microbiome of the Qatari population Relative abundance of the total cohort. Y-axis shows % of relative abundance; X-axis indicates the abundance for Qatari population; each taxonomic category is shown by a different color A) phylum level B) genus level. [file 12967_2020_2291_MOESM1_ESM.jpg]

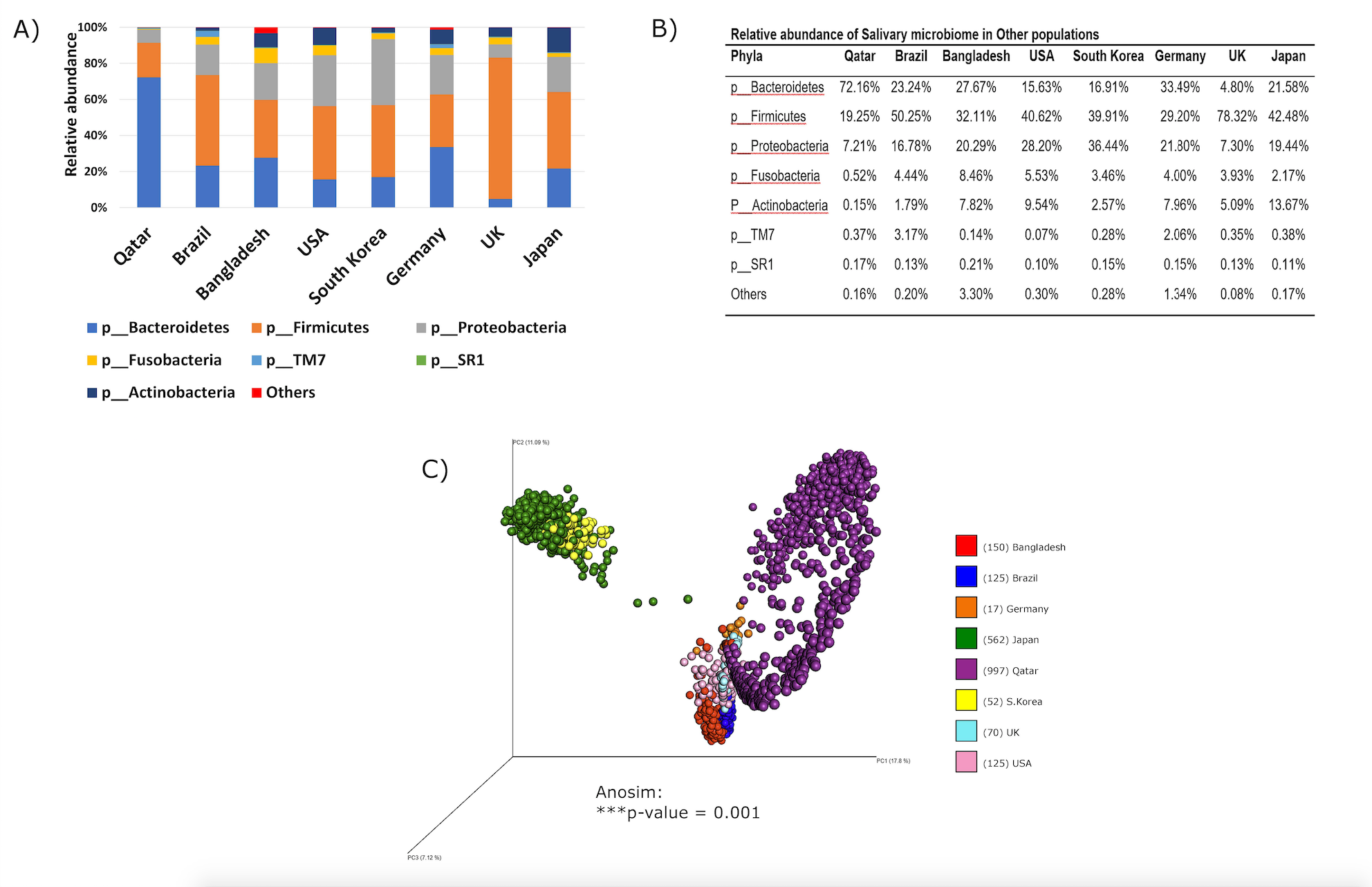

Supplement: Supplementary file 2 — Additional file 2: Figure S2. The salivary microbiome of the other national population. A) Y-axis shows the percentage of relative abundance; X-axis reflects various populations included. Colors in the bar graph reflect bacterial phyla. B) Relative abundance table of the salivary microbiome in various populations at phylum level C) Principle Coordinates Analysis (PCoA) based on Bray–Curtis dissimilarities of the salivary microbiome. Axes are scaled to the amount of variation explained; ***P < 0.001. [file 12967_2020_2291_MOESM2_ESM.png]

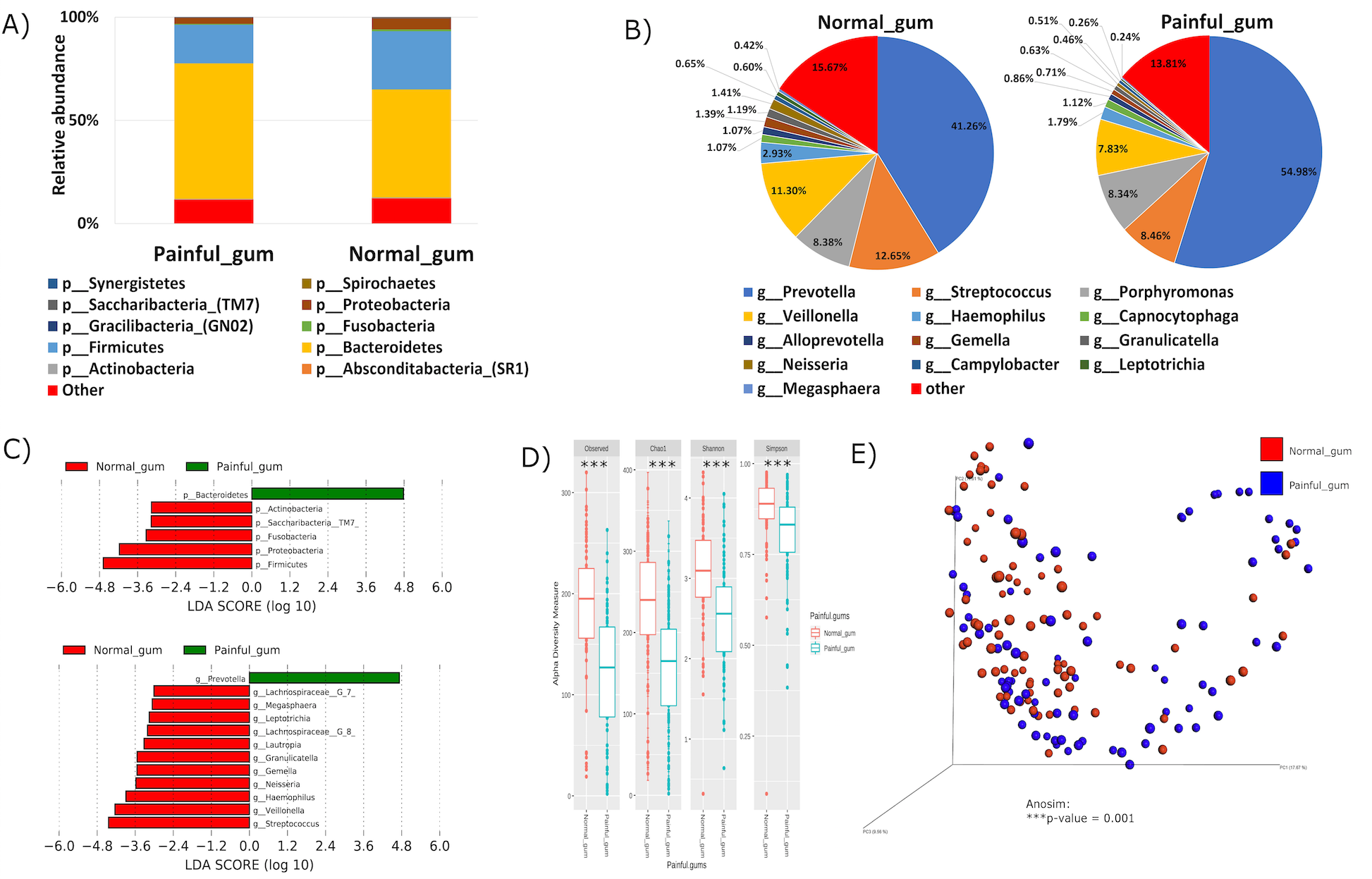

Supplement: Supplementary file 3 — Additional file 3: Figure S3. Salivary microbiome of Participants with Painful gum. Y-axis shows % of relative abundance; X-axis indicates the abundance; each taxonomic category is shown by a different color a) phylum level b) genus level c) Graphs of linear discriminant analysis (LDA) scores for differentially abundant bacterial phyla and genera; among the groups. LDA scores indicate overrepresented bacteria in each group. Features with LDA scores ≥ 2 are presented. d) Alpha diversity measures were used to compare the two groups. Alpha diversity was measured by the number of OTUs observed, by the Chao1 index, in addition to Shannon and Simpson diversity measures, e) Principle Coordinates Analysis (PCoA) based on Bray–Curtis dissimilarities of the salivary microbiome. Axes are scaled to the amount of variation explained; ***P < 0.001. [file 12967_2020_2291_MOESM3_ESM.png]

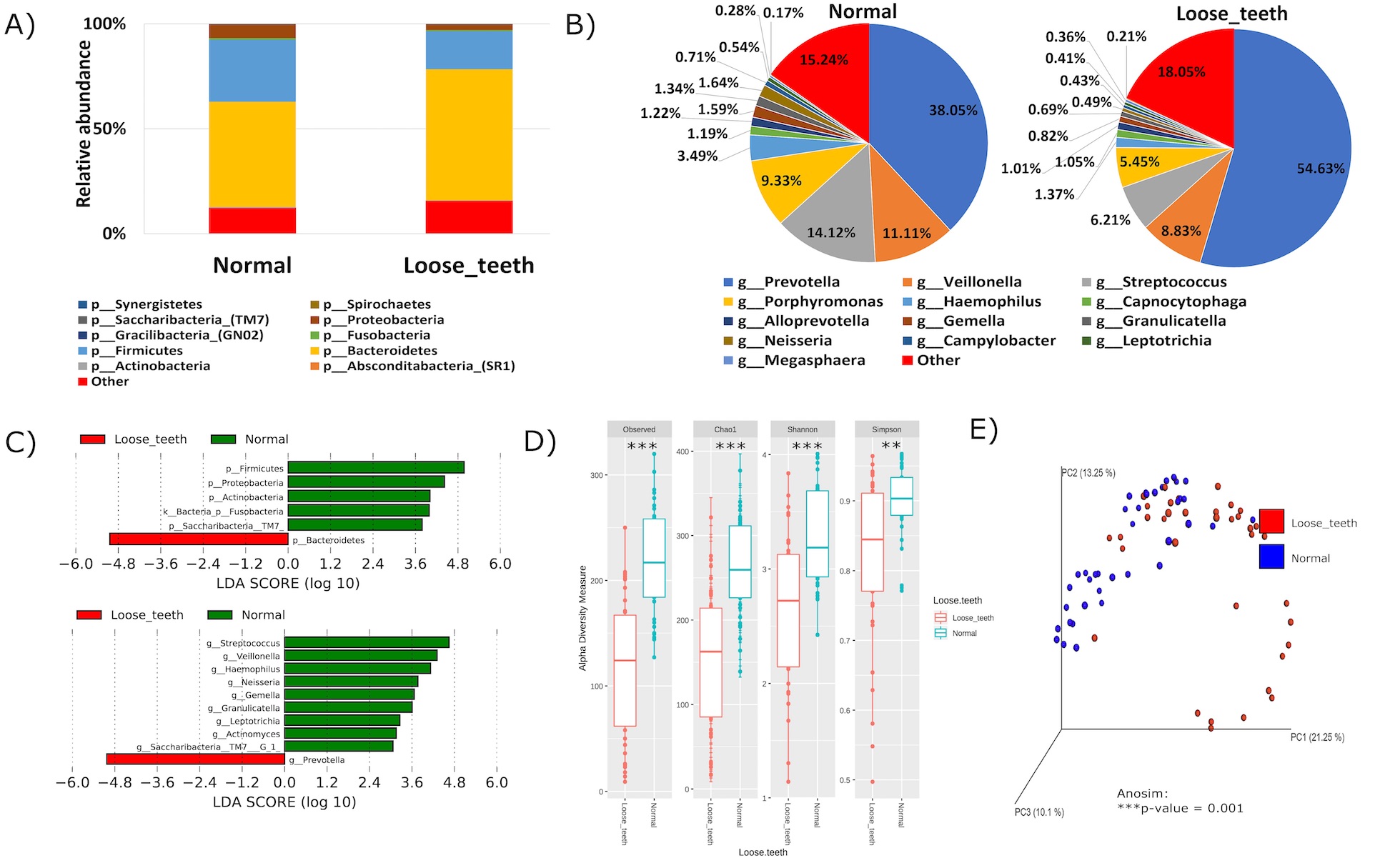

Supplement: Supplementary file 4 — Additional file 4: Figure S4. Salivary microbiome of participants with Loose teeth. Y-axis shows % of relative abundance; X-axis indicates the abundance; each taxonomic category is shown by a different color a) phylum level b) genus level c) Graphs of linear discriminant analysis (LDA) scores for differentially abundant bacterial phyla and genera; among the groups. LDA scores indicate overrepresented bacteria in each group. Features with LDA scores ≥ 2 are presented. d) Alpha diversity measures were used to compare the two groups. Alpha diversity was measured by the number of OTUs observed, by the Chao1 index, Shannon and Simpson diversity measures, e) Principle Coordinates Analysis (PCoA) based on Bray–Curtis dissimilarities of salivary microbiome. Axes are scaled to the amount of variation explained; **P < 0.01, ***P < 0.001. [file 12967_2020_2291_MOESM4_ESM.jpg]
